# Implication of Row Orientation Changes on Fruit Parameters of *Vitis vinifera* L. cv. Riesling in Steep Slope Vineyards

**DOI:** 10.3390/foods10112682

**Published:** 2021-11-03

**Authors:** Timo Strack, Manfred Stoll

**Affiliations:** Department of General and Organic Viticulture, Hochschule Geisenheim University, Von-Lade-Straße 1, 65366 Geisenheim, Germany; Manfred.Stoll@hs-gm.de

**Keywords:** terraced vineyard, berry quality, canopy microclimate, cluster exposition, amino acids, polyphenols

## Abstract

Row orientation, among others, is a crucial factor in determining grapevine performance and health status, thus affecting berry components that form the basis of the later wine profile. However, the literature about the impact of changes in row orientation at steep slope sites on grapevine fruit composition as well as the differentiation between canopy sides hardly exists. Thus, the aim of this work was to gain knowledge about the impact of row orientation in steep slope vineyards on selected primary and secondary metabolites in berries of *Vitis vinifera* L. cv. Riesling. Samples were taken from both canopy sides of different row orientations of terraced and downslope vineyards in steep slopes. Free amino acids in the juice and flavonols in the berry skin had a positive correlation to sunlight exposure. Furthermore, grapevines showed adaptations to constantly higher light conditions, e.g., physiologically in reduction in chlorophyll content or protective mechanisms resulting in a lower susceptibility to sunburn damage. Thus, grapevine fruit parameters are affected by row orientation change in steep slopes.

## 1. Introduction

Content and composition of primary and secondary metabolites present in pulp, skin and seeds of grapevine berries significantly determine wine quality. Furthermore, plants primary metabolites (e.g., sugars, organic acids and amino acids) are crucial for vegetative and generative growth, whilst secondary metabolites (e.g. phenolic, pigment and aroma compounds) play an important role in environmental interactions, e.g., adaptation to biotic and abiotic stressors.

The exposure of grapevine berries to solar radiation and high temperature bears the risk of sunburn and crop damage [1], but is also known to alter the amount and composition of antioxidative substances, which are important for palatability and are valuable for consumers’ health [2]. The ongoing change in climate may, therefore, not only impact the suitability of grapevine cultivation in some regions and its performance, but also berry quality parameters and thus traditional wine profiles [3].

Lately, research was conducted on *Vitis vinifera* L. cultivar Riesling, the leading cultivar for high quality white wine production in Germany and other countries worldwide [4]. Friedel et al. [5] showed that the expression of monoterpene and flavonol metabolic genes in Riesling berries were up-regulated by bunch exposure to sunlight. Further studies confirmed the effect of cluster exposure on the alteration of phenolic substances of berries of the same variety. Sun-exposed bunches resulted in about 50% higher phenolic concentrations on average [6], while shading reduced total phenolics significantly by 43% and 56%, compared to different defoliation means [7]. An increased exposure to UV-B radiation resulted in a decreased content and an altered composition of amino acids in Riesling [7,8], whereas no differences were detected in other grape varieties [9,10,11]. The concentration and composition of certain amino acids is not only associated with the amount of sunlight, daytime and photosynthetic activity of the plant [12], but also depends on grapevine cultivar [13], tissue [14] and origin [15].

Furthermore, the degree of bunch exposure to sunlight is known to affect aroma precursors and compounds of grapes [16]. The impact of natural shading through row orientation on norisoprenoid levels in Riesling was studied in South Africa [17]. Marais et al. [17] found significantly higher norisoprenoid precursors in berries matured under sunlit conditions compared to bunches ripened on the shaded side of the canopy. Since the expression of several aroma compounds in Riesling found to be light dependent [5], it seems that a minimum exposure of above 20% ambient light is necessary for a positive response of monoterpenes [18]. However, the 20% light intensity threshold also applies for the formation of undesired norisoprenoid TDN (1,1,6-trimethyl-1,2-dihydronaphthalene) and its precursor Riesling acetal [19,20].

A change in row orientation, particularly under steep slope conditions, modifies light microclimate in the vineyard [21,22], which affects the diurnal pattern of grapevine canopy temperature, bunch zone light interception [23] and photosynthetically active radiation [24]. Energy input into a vineyard increases with the degree of slope and may result in 30 to 40% higher solar energy intake compared to flat vineyard sites [25,26]. Hence, row orientation changes at steep slopes, i.e., by carving terraces into the slope, affect bunch zone light interception considerably [23].

The aim of this study was to determine the effects of different irradiation conditions on the composition of selected fruit parameters of Riesling grape berries by separating the berry exposition from both canopy sides of different row orientations in two steep slope vineyard systems, i.e., terraced and downsloped vineyards.

## 2. Materials and Methods

In 2019 and 2020, data were collected in the Rheingau wine-growing region on *V. vinifera* L. cultivar Riesling. Three vineyard pairs, each consisting of a vineyard according to the standard cultivation method of vines planted downslope (control, C) and a terraced vineyard system (T) were selected. The down-sloped vineyard site Geisenheimer Rothenberg (GM) represented the N–S row orientation, while the terraced treatment was aligned from E to W. At the sites Lorcher Eisersgrube (LE) and Lorcher Sesselberg (LS) row orientations adapted to the Rhine river-dependent orography. Hence, control vineyards were NE–SW-oriented while terraced vineyards were planted in NW–SE direction. The vines were trained using vertical shoot positioning (VSP), with a single guyot comprising 6–8 buds per m^2^. Row spacing ranged from 1.4 m to 2 m among the control treatment and 1.8 to 2.3 m on terraces, depending on vineyard age and feasibility to mechanization. Vine space ranged from 1 m to 1.2 m in down sloped vineyards and 0.6 m to 0.7 on terraces. Detailed weather conditions can be found in Table S1 and are displayed in Figure S1. Further information on the experimental setup was previously described [23].

### 2.1. Berry Sampling and Processing

At grapevine development stage E-L 38 (berries harvest-ripe [27]), three replicates of one hundred to two hundred healthy berries were randomly collected from each side of the canopy for berry maturity analyses.

For berry skin polyphenol analyses three replicates of twenty berries per sample were cut with the pedicel, flushed with CO_2_ and immediately frozen in liquid nitrogen. Samples were stored at −80 °C until further processing.

#### 2.1.1. Berry Juice Analyses

Berries were pressed twice at 0.6 MPa for approximately two minutes (Longarone 85, Eis System GmbH, Norderstedt, Germany), interrupted by a manual crumbling. The obtained juice was centrifuged for five minutes at 7830 rpm and 20 °C (Eppendorf 5430 R, Hamburg, Germany) and filtered coarsely (33/N). After a second centrifugation step at 14000 rpm for five minutes, the yeast assimilable α-amino nitrogen content was determined by a spectrophotometer (Specord 50 plus, Analytik Jena, Jena, Germany) using a N-Acetyl-L-Cysteine/ophthaldialdehyde (N-OPA) assay [28]. Total soluble solids (TSS), total titratable acidity (TTA), malic acid (MA) and tartaric acid (TA) were analysed by Fourier-transformed infrared spectroscopy (FTIR) via a FT2 Winescan spectrometer (FOSS, Hillerød, Denmark) using an in-house grape must calibration. Amino acid spectrum was obtained using an automatic amino acid analyser S433 (Sykam Chromatographie Vertriebs GmbH, Fürstenfeldbruck, Germany) according to the protocol of Krause and Löhnertz [29]. The separation of amino acids from the juice occurred at a cation exchange separation column via the distinctive isoelectric point of each individual amino acid, aided by a lithium citrate buffer (pH 2.2). Amino acids were qualitatively measured by the retention time. During post column derivatisation, the amino acids were labelled with the reagent ninhydrin at a temperature of 130 °C. An UV/VIS-detector measured the primary and secondary amino acids quantitatively at 570 nm and 440 nm, respectively. All data were transformed from content to concentration (i.e., content per gram berry fresh weight).

#### 2.1.2. Berry Skin Polyphenol Analysis

Frozen berries were peeled under CO_2_ atmosphere. Berry skins were freeze dried, ground and desiccated until further processing. Phenolic compounds were then extracted by acidified acetonitrile under SO_2_ protection prior to vacuum distillation. The polyphenolic extracts were analysed by an UHPLC system (UltiMate 3000, ThermoFischer, Dreieich, Germany) coupled to a UV/VIS diode array detector (Vanquish, ThermoFischer, Dreieich, Germany). Additionally, samples were measured by a LXQ mass spectrometer (ThermoFischer, Dreieich, Germany). Then, 3 µL sample volume were injected at a flow rate of 250 µL min^−1^ into a 150 × 2 mm (inner diameter) 3 µm Luna 3u C18 100 A column (Phenomenex, Aschaffenburg, Germany) for chromatographic separation. Flavanols, phenolic acids and flavonols were detected at wavelengths of 280 nm, 320 nm and 360 nm, respectively. The identification of the peaks was based on HPLC retention time, the UV spectra and mass spectral data. External calibration curves were used for quantification.

Elution conditions were: solvent A was 2% acetic acid; solvent B was acetonitrile/ water/acetic acid (50:50:0.5; *v*/*v*/*v*). Gradient elution was applied: 0–20 min from 96–50% solvent A, 4–50% solvent B, 20–23.1 min to 100% B; washing with 100% B for 2 min before re-equilibrating the column. The following mass spec conditions were used: ESI source voltage −3.00 kV during negative and +5.00 kV during positive ionization mode; capillary temperature 275 °C; collision energy for MSn-experiments 35% (arbitrary units). A table containing all standard sources is presented in the supplementary material (Table S3). Where no standards were available, substances were quantified using the calibration for the closest phenolic relatives (caftaric acid as caffeic acid; fertaric acid as ferulic acid, coutaric acid and p-CGT as coumaric acid) [7].

### 2.2. Canopy Density Measurements

Point Quadrat Analysis [30] was conducted at flowering, onset of ripening and at harvest to obtain additional information on bunch zone light conditions. Data was collected by inserting a thin metal rod into the canopy along a measuring tape. Distance between insertions was 20 cm. One hundred insertions were made within four replicates. Leaf layer number (LLN), percentage of interior clusters (PIC) and percentage of interior leaves (PIL) were calculated from recordings of contact with leaves and clusters, according to Smart and Robinson [30]. The data is presented in the Supplementary Material (Table S4).

### 2.3. Cluster Light Interception

Cluster light environment was measured via dyed triacetyl cellulose strips (OptoLeaf R-3D, Taisei-Environmental & Landscape Group, Tokyo, Japan). A grid (1 m × 0.3 m) equipped with fifteen light sensitive films (LSF) was placed in front of either canopy sides, both sunlit and shaded, at bunch zone height (0.7 m to 1 m) at all experimental sites in 2019 and 2020 during same time intervals at flowering (LSF_f), onset of ripening (LSF_o) and berries harvest-ripe (LSF_h). Film fading values were converted by calibration curves via regression equations obtained from a nearby weather station, maintained by Hochschule Geisenheim University (49°98′4″ N; 07°95′44″ E) [23]. The data were used as additional quantitative parameter in principal component analysis to incorporate environmental data, which is an important driver of grapevine fruit quality. The data are presented in the supplementary material (Table S5).

### 2.4. Leaf Nutritional Status

A functioning photosynthesis apparatus is important for plant growth in general and is determining for yield and berry quality in grapevine, since assimilated carbon is a key element in organic structures such as carbohydrates, amino acids or polyphenols. A chlorophyll-meter (Dualex 4 scientific, Force-A, Orsay, France) was used for leaf chlorophyll measurements at two dates (flowering and bunch closure). The optical chlorophyll index (Chl_i_) was obtained by measuring the abaxial and adaxial side of three leaves per canopy side on three selected and labelled shoots of six randomly selected vines of each treatment and site. Leaf position and leaf age was considered similar due to measuring at comparable internode lengths. The Chl_i_ index is recognized to perform well as a proxy for leaf nitrogen content [31].

### 2.5. Scoring of Sunburn Damage

Subsequent to a row of heat days (i.e., daily maximum temperatures >30 °C), sunburn damage was assessed in both years of 2019 (01.08.) and 2020 (13.08.). Four hundred grape cluster per vintage, site, treatment and canopy side were evaluated following the seven steps assessment scheme of EPPO guideline 1/031(3) [32], in order to evaluate reactions of clusters exposed to different light regimes under extreme environmental conditions.

### 2.6. Statistical Analyses

Principal component analysis (PCA) was used for exploratory data analysis of berry ingredients to examine the associations between individuals and variables and to detect main components for later analyses. Data were checked for correlations and auto scaled (i.e., $\frac{xi-mean(x)}{sd(x)}$). PCA was performed using *R* packages *FactoMineR* [33] for conducting and *factoextra* for visualization. All analyses were implemented within the *RStudio* environment (v1.4.1106 (11 February 2021)).

Pairwise multiple comparison via Student–Newmann–Keuls test (significance level of α = 0.05) was applied subsequent to one-way analyses of variance (ANOVA) conducted on data of berry juice (harvest parameters and amino acids) and berry skin (polyphenol analyses) as well as Chl_i_ measurements for the factor exposition. Subsets were divided into single vintages and in case of Chl_i_ measurements into development stages. All data sets were tested for homoscedasticity of variance via Levene test and checked visually for normal distribution of residuals.

Sunburn data were analysed via a non-parametric Kruskal–Wallis test. Results were compared post hoc via Dunn’s test (Bonferroni-adjusted, significance level of α = 0.05).

Statistical analyses were conducted using *R* packages *agricolae* [34], *car* [35] and *multcomp* [36].

## 3. Results

**Figure 1:**
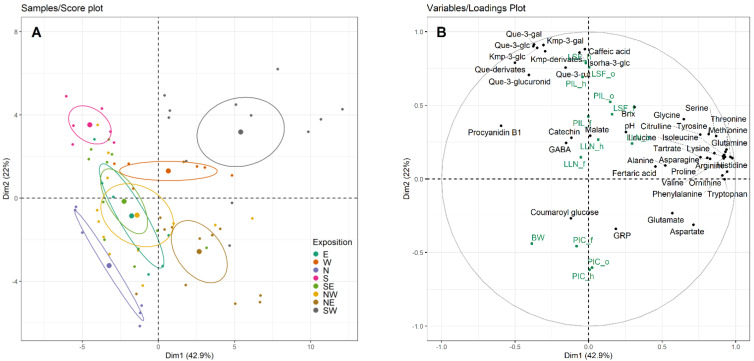
Principal component analysis. (**A**) Score plot of individuals. Dots represent individual samples and dot colour indicates bunch exposition. Big dots show the mean of sample group, ellipses represent 95% confidence interval. (**B**) Loadings plot of variables. Black labels show analysed berry parameters (concentrations). Green labels represent supplementary quantitative data. BW = berry weight, LLN = leaf layer number, PIC = percentage of interior clusters, PIL = percentage of interior leaves (_f = flowering, _o = onset of ripening, _h = harvest-ripe), LSF = bunch zone radiation energy interception, GABA = γ-aminobutyric acid, gal = galactoside, glc = glucoside, rut = rutinoside, GRP = grape reaction product, Kmp = kaempferol, Que = quercetin.

Principal component analysis explained approximately 65% of total variance. In total, 72 individual samples and 54 variables (i.e., six technological juice parameters, 28 amino acids and related derivates as well as twenty polyphenolic compounds) were analysed by principal component analysis. Additionally, thirteen quantitative variables (i.e., cluster light environment data, canopy density parameters and berry weight) were added. The best distinguishing factor for the individual samples was (bunch) exposition. The score plot (Figure 1A) illustrates the individual samples grouped by exposition. Figure 1B displays the variables with the highest contribution.

SW-exposed canopy sides were characterized by a positive coordinate on the first dimension (Dim1) and the second dimension (Dim2) axes (Figure 1A). SW exposition of terraced vineyards showed high association with amino acid concentrations and low values of berry weight, procyanidin B1 and PIC (Figure 1B). Whereas S-exposed berries showed a strongly negative association with Dim1 (Figure 1A), thus low levels of amino acids. Dimension 2 opposed individuals of S- and SW-exposition (strong positive correlated to the axis) to individuals of N- and NE-exposition (strong negative relationship to the axis). A positive association to Dim2 related to bunch zone sun light interception (LSF) and flavonols.

Distance between sunlit (i.e., S, SW) and shaded (i.e., N, NE) sides of terraced vineyards (Dim2), but also between row orientations, in particular of terraced vineyards (Dim1), and exposition, was apparent. The canopy sides of control vineyards (E, W, NW, SE) scattered around the centre with a small negative association to Dim1. The W- and SE-exposed berries showed a trending positive association to Dim2 (Figure 1A), demonstrating their higher share on daily radiation compared to the E- and NW-facing canopy sides.

### 3.1. Berry Composition

To evaluate the effect of irradiation, harvest-ripe berries were picked from the different canopy sides and analysed separately.

#### 3.1.1. Berry Juice Composition

Analysed parameters of juice obtained from harvest-ripe berries showed similar trends between the two vintages 2019 and 2020 for sites with the row comparison of NE–SW and NW–SE (sites LE and LS). Control vineyards achieved higher contents of total soluble solids and berry weight compared to the terraced treatment, resulting in a lower concentration of TSS (Table 1). The highly sun-exposed canopy sides W and SE showed the highest Brix values per gram berry weight.

Amounts of tartrate and malate were low for SE-exposed berries, resulting in lowest amounts of total titratable acidity, along with the NW-exposed samples. Terraced vineyards in NW–SE orientation showed the highest levels of acidity, but tended to result in the highest pH (Table 1). The E–W-oriented terraced treatment achieved higher amounts of total soluble solids and berry weight compared to the N–S-oriented control. TTA was highest for control treatments in 2019, but similar between treatments in 2020. In general, the hill-facing canopy side of the terrace (N) and the shaded side of the control vineyard (E) showed the highest amounts of malate within this site. The juice pH mainly increased on the W-exposed canopy side (Table 1).

N-OPA values were the highest in samples of SW- and NE-exposed berries. Here, the more sunlit canopy side (SW) showed higher values than the hill-facing NE-exposed side. Samples from NE–SW oriented control vineyards had lower N-OPA values than the terraced vineyards. In N–S0-oriented vineyards, the sunlit W-exposed berries showed higher N-OPA concentrations than E-facing samples, while both canopy sides of the terraced treatment did not differ in their low N-OPA concentrations (Table 1).

**Figure 2:**
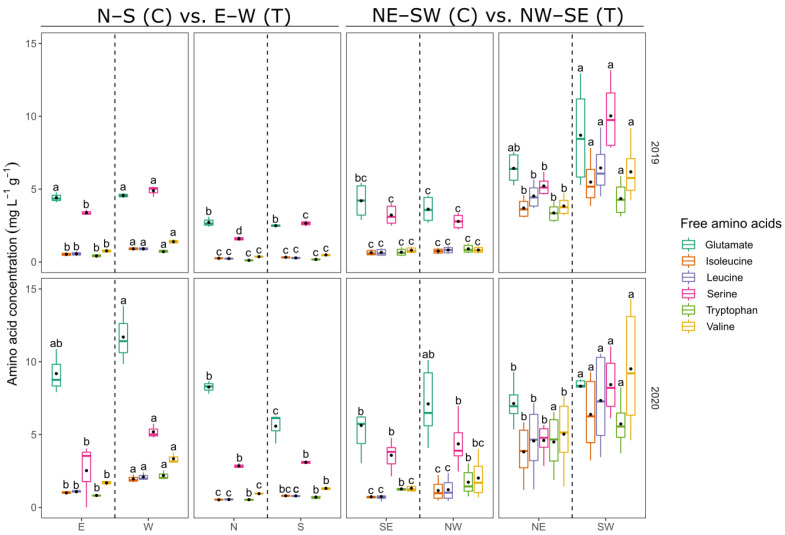
Comparison of selected primary amino acid concentrations (mg L^−1^ g^−1^) obtained from juice of harvest-ripe berries. Boxplots show the distribution of the data. Median is indicated by horizontal line in the boxplot, mean values are represented by a black dot in the respective boxplots. Different colors indicate different amino acids. Lower case indicates results of Student–Newman–Keuls post-hoc test (different letters show significant differences (*p* < 0.05)) between canopy exposition (E = east, W = west, N = north, S = south, SE = southeast, NW = northwest, NE = northeast, SE = southeast) within row orientation pairs in respective vintage.

Treatments were significantly different in amino acid concentrations (Table S6). Similar trends among different row orientation systems were observed. In both years, the N–S-oriented control vineyard showed higher free and total amino acid concentrations compared to the terraced treatment (Table S6). The E-exposed berries of the control usually showed lower free amino acid concentrations compared to the more sunlit W side of the canopy (Figure 2). The hill- and valley-facing sides of terraced vineyards planted in E–W orientation (N and S) showed no differences in the total concentration of amino acids.

However, terraced vineyards planted in NW–SE direction contained higher amounts of total and single amino acid concentrations compared to NE–SW oriented control vineyards (Figure 2, Table S6). The NE–SW control vineyard often showed similar values of single and total amino acid concentrations of grape juice obtained from NW- and SE-exposed berries (Figure 2, Table S6). In NW–SE-aligned vineyards (T), SW-exposed berries usually showed higher amounts of single free amino acids compared to NE-exposed samples (Figure 2).

The proportional difference of total amino acids between SE (stronger light exposed) and the, within the course of a day, less sun-exposed NW side of the canopy ranged between −2.63% and 52.14%, resulting in no significant differences (Table S7). Row orientation change to NW–SE alignment increased the differences in total amino acids to 1.8- to 4-fold (NE) and 2.2- to 6-fold (SW), depending on the year and site. W-facing canopy sides showed about 1.5 times higher total amino acid concentrations compared to the E-facing side. N- and S-exposed berries (T) showed approximately half of the total amino acid content compared to the comparative exposition E (C).

Arginine, proline and glutamine were the most abundant free amino acids in terms of proportion (total sample averages: 25%, 19% and 12%, respectively; Table S7). Arginine was found in high concentrations at site LE and LS in both treatments and years. Terraced vineyards at site LE and LS showed higher concentrations for arginine and glutamine compared to control. In turn, control vineyards planted in NE–SW direction showed higher concentrations and share of proline (Tables S6 and S7). Proline was also higher in terraced vineyards of E–W orientation, while arginine and glutamine were more abundant in the N–S-aligned control vineyard. Glutamate, GABA and alanine had a maximum share of 13.5% on total amino acid concentration. Glutamate, GABA and alanine showed higher concentrations in berries from NE–SW oriented terraces compared to control, but had a lower share on total amino acids. Vines planted in N–S direction showed higher values in glutamate, GABA and alanine, but the values represented a lower proportion to total amino acid concentration compared to the E–W-aligned terraced treatment (Tables S6 and S7).

#### 3.1.2. Berry Skin Polyphenol Content

**Figure 3:**
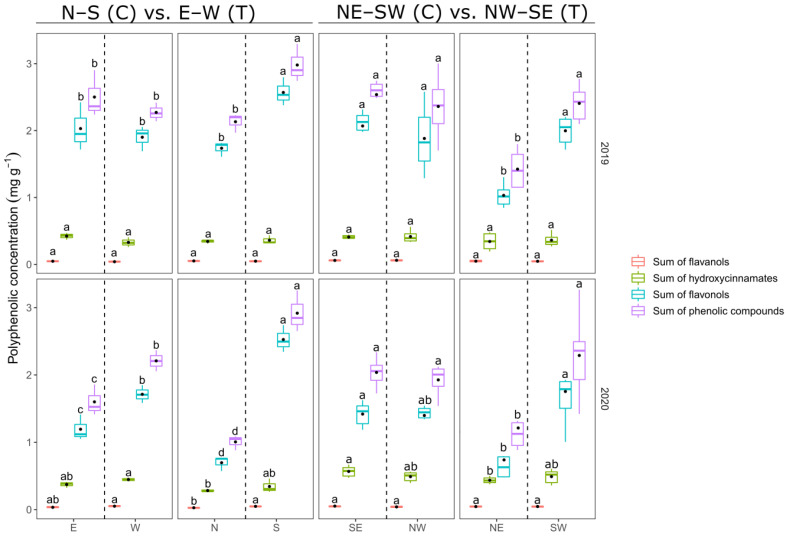
Comparison of sums of polyphenolic concentrations (mg g^−1^) obtained from skins of harvest-ripe berries. Boxplots show the distribution of the data. Median is indicated by horizontal line in the boxplot, mean values are represented by a black dot in the respective boxplots. Different colors indicate different groups of polyphenolics. Lower case indicate results of Student–Newman–Keuls post-hoc test (different letters show significant differences (*p* < 0.05)) between canopy exposition (E = east, W = west, N = north, S = south, SE = southeast, NW = northwest, NE = northeast, SE = southeast) within row orientation pairs in respective vintage.

Results of berry skin polyphenols were consistent during both years of the experiment. Only single parameters showed a little variation between vintages. In general, catechin and procyanidin B1 did not differ much among exposition and sites. However, control vineyards showed a tendency of higher values for the sum of flavanols (Figure 3, Table S8).

At site GM, the W-exposed berries showed highest amount of fertaric acid and caffeic acid in 2020, while N- and NE-exposed berry skin extracts showed the lowest values consistently. Nevertheless, differences in results of total hydroxycinnamates were not consistent between the years (Table S8).

Flavonols were highest for berries harvested from the valley-facing sides of the terraced vineyards (S and SW). SE- and NW-exposed samples from the control vineyards did not differ, while W-exposed berries tended to have higher flavonols and sum of phenolic content compared to those exposed to the east (Figure 3, Table S8). In essence, the more sun-exposed canopy sides showed the highest values in sum of phenolic content of the berry skin (Figure 1A,B and Figure 3).

### 3.2. Leaf Nitrogen Content

**Figure 4:**
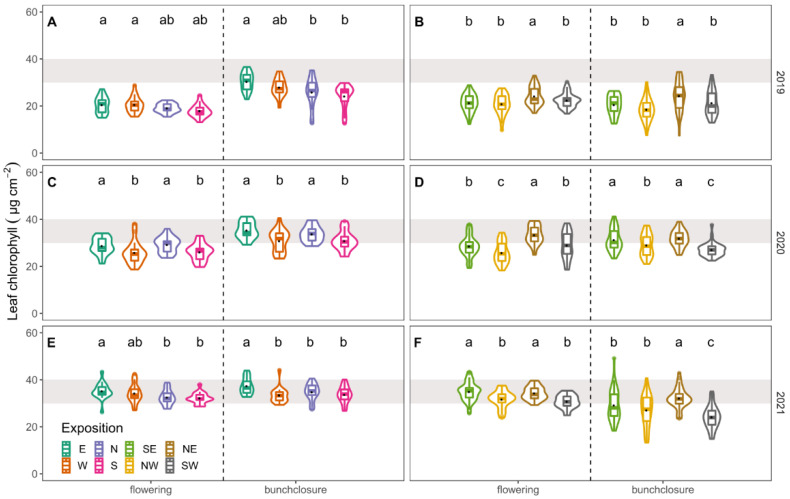
Leaf chlorophyll content (µg cm^−2^) measured at development stages flowering and bunch closure in 2019 (**A**,**B**), 2020 (**C**,**D**) and 2021 (**E**,**F**). Violins show the distribution of the data. Median is indicated by horizontal line in the boxplot, mean values are represented by a black dot in the respective boxplots. Different colours represent different canopy expositions. The grey background shows approximated optimum range [31]. Lower case indicate results of Student–Newman–Keuls post hoc test (different letters show significant differences (*p* < 0.05)) between canopy exposition (E = east, W = west, N = north, S = south, SE = southeast, NW = northwest, NE = northeast, SE = southeast) in respective vintage and development stage for N–S/E–W and NW–SE/NE–SW row orientations.

Significant differences (*p* < 0.05) were found for leaf Chl_i_ measurements between expositions on each measurement date (Figure 4). Single canopy sides did not always reach optimum Chl_i_ content [31] during the three years tested. Mainly NW- and SW-exposed canopy sides, but also S- and W-facing leaves showed the lowest, and often insufficient, leaf Chl_i_ values.

Results of Chl_i_ measurements were different for the respective canopy side comparisons between the vintages. Chl_i_ content increased in N–S (control) and E–W (terraced) oriented rows from flowering to bunch closure in all years. The Chl_i_ content of the control vineyard was higher compared to the E–W-oriented terraced treatment (Figure 4A, C), while in 2020 there was no difference between treatments. In 2021 Chl_i_ values were highest for the observed canopy sides. Again, control vineyards tended to show higher leaf chlorophyll content compared to the terrace, mainly due to high mean value of E-facing leaves (Figure 4E).

NW–SE oriented terraces showed higher Chl_i_ values at flowering compared to the control vineyards planted in NE–SW direction (Figure 4B, D). In 2021, data were less clear due to high Chl_i_ levels in SE-exposed leaves (Figure 4F). At bunch closure, the NE–SW aligned control vineyard tended to have a higher mean leaf Chl_i_ content. In general, less sun-exposed canopy sides (E, N, NE, but also SE) showed higher Chl_i_ values compared to their sun-exposed equivalents, which had a higher sun exposition during the afternoon and early evening (S, W and NW and SW).

### 3.3. Sunburn Damage

**Figure 5:**
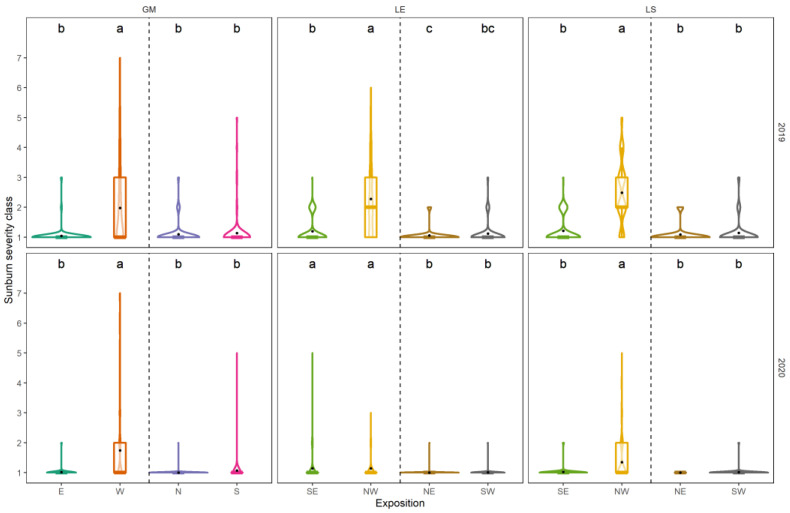
Post-heat wave sunburn damage scoring for vintages 2019 and 2020 for three different sites (Geisenheimer Rothenberg = GM, Lorcher Eisersgrube = LE, Lorcher Sesselberg = LS). Treatments are separated by the vertical dashed line. Violins show the distribution of the data (n = 400). Median is indicated by the horizontal line in the boxplot, mean values are represented by black dot in the respective boxplots. Different colours represent different canopy expositions. Lower case indicate results of Dunn’s test (different letters show significant differences (*p* < 0.05)) between canopy exposition (E = east, W = west, N = north, S = south, SE = southeast, NW = northwest, NE = northeast, SE = southeast) at the respective site, vintage and development stage for N–S/E–W and NW–SE/NE–SW row orientations. Definition of sunburn severity classes: 1 = no disease; 2 = < 5%; 3 = 5–10%; 4 = 10–25%; 5 = 25–50%; 6 = 50–75%; 7 = > 75% (according to EPPO guideline PP 1/031(3)).

The results of sunburn-damaged grapes are shown in Figure 5. In general, sunburn damage was higher in 2019 compared to the vintage of 2020.

At the site GM, the W-exposed side of the canopy showed a significant higher severity of sunburn damage on the grapes compared to the E-exposed side of the same canopy or any canopy side of the terraced vineyard (Figure 5). However, the during the day constantly irradiated valley facing side of the terrace (S) did not differ from the shaded canopy sides facing N or E.

In both years, control vineyards planted in NE–SW direction showed higher incidences of sunburn-damaged grapes compared to terraced vineyards in NW–SE alignment. The most affected treatment was the control with its NW-exposed canopy side, followed by the sunlit SE-exposed canopy side (Figure 5). Small sunburn incidences were observed at SW-facing canopy sides of terraced vineyards, in spite of higher energy input. The hill-facing NE-exposed canopy side was not affected by sunburn damage.

## 4. Discussion

Recent research described the microclimatic conditions of two management systems with different row orientations in steep slopes [23]. It was found that daily temperature parameters were generally higher in steep slope vineyards planted downslope compared to terraced vineyards, especially for N–S row orientations, except for daily minimum temperature. While the valley-facing sides of terraced vineyards showed the highest light interception values at bunch zone height, canopy sides of downslope aligned vineyards were more balanced in sun exposure [23]. Grapevine fruit quality crucially depends on microclimate, mainly described by berry exposition to the sun [37]. Slightly excessive light and temperature leads to metabolic protective mechanisms, e.g., accumulation of phenolics or heat shock proteins, known to alter later wine quality [38,39,40]. Previous research conducted on flat vineyards focused on differences in microclimate [41,42], physiological behaviour of the grapevine [43,44] and its effects on fruit parameters [45,46] and wine [47,48,49] in respect to row orientation changes, further considering differences in canopy sides. However, to our knowledge, literature about the impact of row orientation on steep slopes on grapevine fruit composition as well as a differentiation between canopy sides is not available. Thus, data presented in this work will help to understand the role of quality determining factors such as row orientation and its impact on primary and secondary metabolites of *V. vinifera* L. cv. Riesling in steep slope vineyards.

### 4.1. Berry Parameters

It needs to be considered that vineyards were cultivated by different wineries with individual schedules of vineyard management. Despite the renunciation of fertilizers during the experimental years, an influence of the vineyard management cannot be excluded, i.e., by different timing of, e.g., soil or canopy management. Nonetheless, applied means followed the common practice and is partly owed to the management system itself.

Beyond this, a clear distinction of canopy sides, row orientation and treatments was shown by the principal component analysis (Figure 1A).

#### 4.1.1. Berry Juice Parameters

Related to berry weight, total soluble solid values were always the highest in SW-exposed berries and higher in S- and W-exposed berries compared to N- or E-facing berry samples (Table 1). This is based on a concentration effect, coinciding with smaller berries due to higher light exposure [50]. The concentrations of organic acids were usually highest in terraced vineyards planted in NW–SE direction and were also generally higher for small berries, predominately from well-irradiated canopy sides, e.g., W and SW (Table 1).

The yeast assimilable nitrogen content, expressed as N-OPA, was generally low in both years, due to multiannual drought stress of previous seasons 2017 to 2019. A sufficient provision of yeast assimilable nitrogen, e.g., ammonia and primary amino acids, may not necessarily lead to more aromatic wines [51], but guarantees growth of *Saccharomyces cerevisiae* and a successful fermentation [52]. The concentrations of N-OPA and amino acids varied between treatments of different row orientations. In both years, differences between sunlit and shaded canopy sides existed at each site within the same vineyards and the same row orientations (Table S6). Total amino acid concentrations were always significantly higher in W-exposed berries, compared to those picked from the E-exposed side of the control canopy. Additionally, grape berries harvested from the valley-facing side (SW) of NW–SE oriented terraces showed significantly higher total amino acid concentrations compared to the hill-facing NE-pendant. Beside the fact that no differences were found among total amino acid concentrations in the comparison of N- to S-facing berries on terraces or NW- to SE-exposed control berries (Table S6), juice from sun-exposed berries showed higher amino acid concentrations compared to those derived from shaded berries. This is in contrast to earlier studies on Riesling relating the inhibition of amino acid synthesis to sunlight UV-B [7,8,11], but is in accordance with the findings in other grapevine cultivars [9,14]. Bunch zone leaf removal decreased amino acid concentration significantly in Sauvignon blanc [53]. The application of leaf removal on the shaded, i.e., less sunlit, side of the canopy is a common practice in viticulture to enhance aroma precursors and to prevent diseases. Bunch zone leaf removal may lower amino acid concentration due to a reduction in source leaves. In 2019 leaf removal was applied moderately at site GM at both treatments (C and T) on the shaded sides of the canopy (E and N, respectively). Whilst the E-facing berries of N–S-oriented rows showed a significant decrease (−28.5%) in total amino acid concentration compared to the more sunlit, non-defoliated, W-side of the canopy, there was no difference between the canopy sides of the E–W-oriented terraced vineyard (Table S6). A higher number of leaf layers (LLN, Table S4) may explain why the highly sun exposed S-facing canopy side of the terrace did not differentiate from the hill-facing N-side. Solar radiation is highly absorbed by single leaf blades, leading to an effective shading of subjacent leaves or clusters [30]. However, during berry ripening, the maximum temperature of both canopy sides of E–W-oriented vineyards coincide in the early afternoon [23], leading to the assumption that the contradictory role of UV-B on amino acids may play a subordinated role to temperature. Another possible explanation might be the compensation capacity of grapevines shown after defoliation treatments, by which photosynthetic activity is regained theoretically [54]. LLN was low at the SE-facing side of the canopy (Table S4). During the day, radiation may have penetrated the porous canopy side and warmed up NW-facing clusters. S-, W- and SW-canopy sides constantly showed warmer temperatures during the day, while SE- sides of control vines are just slightly more favoured by sunlight than the NW-sides [23]. Leaves shading the clusters may reduce excessive heat leading to a more favourable condition for amino acid accumulation in the berries. Nevertheless, Arrizabalaga-Arriazu et al. [55] found a tendency to the reduction in total amino acid concentration at maturity for a temperature difference of 4 °C compared to ambient environment, whereas the relative abundance of aspartates, shikimates, phosphoglycerates and pyruvates was increased. However, results of the study showed high standard errors, reducing the significance. GABA, proline and their precursor glutamate were increased in berries of heated vines [56] and were attributed to warm growing seasons [57]. Same amino acids were also elevated in samples from commonly warmer N–S-oriented control vineyards and terraces in NW–SE orientation in this study, where highly exposed SW-canopy sides reached high maximum temperatures [23]. It was suggested, that GABA plays a role as a protective against UV-B-induced oxidative stress in grape berries [11]. Furthermore, amino acid biosynthesis of several amino acids was up-regulated under drought stress conditions [58,59]. Proline, leucine, isoleucine, and valine as well as serine and tryptophan were elevated for highly exposed S- and SW-facing canopies (Figure 2). Therefore, it can be concluded, that highly sun-exposed berries responded to the stressors of light and high temperatures, possibly accompanied by drought stress, by accumulating some of the defence-related amino acids.

Contrarily to others [38,60], samples derived from elevated temperature and high light influenced SW-sides of the canopy were high in above mentioned amino acids, but also showed high values of malate (Table 1). Commonly, low berry malate content is attributed to high temperature exposure post-véraison [61,62]. However, Sweetman et al. [56] found that berries exposed to higher temperatures post-véraison decreased in malate content, but did not change when minimum temperatures were also elevated. Higher values of malate from berries of the highly sun-exposed SW side of the canopies may be explained by generally higher night temperatures in terraced vineyards [23].

#### 4.1.2. Berry Skin Parameters

Flavonols showed a strong correlation to light exposure (Figure 1B). Pieri et al. [46] defined a positive relationship between incoming solar radiation at berry scale with the total amount of berry skin flavonols. Flavonols were proposed to be an adequate indicator for differences in light microclimate [40,63]. Martínez-Lüscher et al. highlighted the correlation between Que-3-glc and Kmp-3-glc with the summed amount of UV-B received by Tempranillo clusters [11] and Friedel et al. [7] found no increase in quercetin glucosides in shaded Riesling clusters. In this study, those two flavonols were lowest in sunlight-reduced expositions of the terraced canopies (namely N and NE; Table S8), where the canopy light regimes are only one fifth of global radiation [23]. No clear effect of cluster exposition could be attributed to the sum of flavanols and total hydroxycinnamates (Figure 3), but caffeic acid and fertaric acid tended to be higher in more sunlit samples, approving previous findings [5,6,7,38]. The opposed behaviour of flavonols and hydroxycinnamates was also shown by Reshef et al., who highlighted the importance of canopy side and berry orientation on grape berry metabolic composition due to light intensity levels [38,60]. While polyphenols play a major role in red wine production, too high concentrations in juice and wine of white grape cultivars may be detrimental to consumers’ perception. Therefore, cluster exposure to high light intensities and temperature must be well considered concerning desired wine style. Furthermore, berry health usually benefits from light exposure, but berries are vulnerable to solar heating resulting in loss of berry mass and quality or sunburn damage [1,64].

Sunburn is the physiological disorder which occurs on berries after the exposure of intensive irradiation by sunlight, due to high UV radiation and excessive temperature [1].

Sunburn scoring data clearly showed, that the during the afternoon exposed canopy sides (i.e., W and NW) of a grapevine are more prone to damage compared to berries growing in less sunlit locations of the plant, i.e., E, N and NE, (Figure 5). Interestingly, the most sun-exposed canopy sides (S and SW) did not differ from their shaded equivalent (i.e., N and NE). Several authors pointed out the importance of row orientation and, thus, exposition, on grape sunburn damage due to high irradiation of the afternoon sun [1,65,66]. Apart from this, grapevines are able to acquire berry protection due to several mechanisms [1]. Light induces photo-protective mechanisms, including the metabolism of phenolic components [5,67] or heat shock proteins [68,69], both relying on sufficient amino acid resources, e.g., phenylalanine. Phenylalanine was high in exposed S-, W- and SW-berries (Table S6). The role of phenylalanine in secondary metabolite biosynthesis is crucial, due to its key role in the phenylpropanoid pathway [70]. Furthermore, phenylalanine is explicitly rate-limiting for some secondary metabolites in grapevine, e.g., quercetins [71]. Samples high in the amino acid phenylalanine were also high in flavonols, such as several quercetins (Tables S6 and S8). While SW-exposed samples showed the highest concentrations of stress responding amino acids (Table S6), S-facing berries showed the highest values of sun-screening flavonols (Table S8). The increased incidence of sunburn on W- and NW-facing clusters was also promoted by the wave angle of sunbeams. The lower sun position leads to a more directed warming of the clusters, which does not affect S-facing canopy sides during noon, when sun’s zenith is reached. Consequently, row orientation changes successfully prevented sunburn damage (Figure 5).

### 4.2. Leaf Nitrogen

Light stimulates aboveground growth of the grapevine [72]. A higher exposed leaf mass under non-limiting growth conditions benefits carbon assimilation. However, excessive light conditions bare the risk of damaging proteins and the light harvesting pigments of the photosynthetic system [73]. High irradiation of leaf tissue results in significantly lower leaf chlorophyll content compared to shaded leaves [74,75], which was also confirmed for exposed canopies in this study (Figure 4). Suggested optimum values for leaf Chl_i_ content were seldom reached by the different canopy sites of the examined steep slope management systems [31]. N-, E- and NE-exposed leaves showed the highest Chl_i_ values (Figure 4). However, under humid conditions of 2021, leaves of the W- and S-side of the canopy showed optimum Chl_i_ values. This highlights the importance of water availability for leaf morphology and thus photosynthetically performance and can be used as a proxy for the estimation of leaves physiological and nutritional status [76], further estimating grapevine performance [77]. In this study, however, it is not possible to distinguish between performances of single canopy sides using Chl_i_ content as a proxy. Although, north-, east- and northeast-exposed canopies showed highest chlorophyll levels, shaded canopy sides performed inferior to more sunlit canopy sides (Figure 4, Table 1). While it is possible to influence performance of source organs [78], the partitioning of carbon structures (i.e., sucrose and amino acids) follows physical (sink gradients) or enzymatic regulatory mechanisms in the berry [79].

## 5. Conclusions

In steep slope vineyards, fruit composition was affected by a change in row orientation mainly due to microclimatic effects of sunlight (i.e., irradiation and temperature). Additionally, water supply may play a major role in nutrition availability and grapevine performance. Whilst terraced vineyards reduce the risk of erosion and improve the infiltration during precipitation, not fully adapted vines suffer from water stress are likely to be hassled further through a higher evapotranspiration demand from a higher exposed surface of the embankments. Hence, further research must evaluate applicable methods to improve soil water retention, grapevine root development and hydraulics in steep slope vineyard systems.

A change in cluster exposition, as a consequence of a change in row alignment, showed to be a viable tool to reduce the risk of sunburn. Furthermore, the differences in fruit composition between canopy sides of terraced vineyards may be useful to achieve different quality goals in view of climate change adaption, i.e., lower TSS or maintaining higher acidity, by selective harvesting.

## Figures and Tables

**Table 1:** Berry maturity parameters (mean ± standard deviation) of Riesling juice obtained from harvest-ripe berries in 2019 and 2020, separated by canopy sides of each treatment (control = C, terraced vineyard = T). Lower case indicates results of Student–Newmann–Keuls test. Different letters show significant differences (*p* < 0.05) between canopy expositions in respective vintage for every site. GM = Geisenheimer Rothenberg, LE = Lorcher Eisersgrube, LS = Lorcher Sesselberg, TSS = total soluble solids, TTA = total titratable acidity, N-OPA = yeast assimilable nitrogen. Table shows juice parameter content per gram berry fresh weight.

Vintage	Site	Treatment	Exposition	TSS (°Brix g^−1^)	TTA (g L^−1^ g^−1^)	Tartaric Acid (g L^−1^ g^−1^)	Malic Acid (g L^−1^ g^−1^)	pH	N-OPA (g L^−1^ g^−1^)	Berry Weight (g)
2019	GM	C	E	22.50 ± 0.44 a	12.00 ± 0.67 a	9.46 ± 0.52 a	3.18 ± 0.20 a	2.90 ± 0.00 b	60.06 ± 3.97 b	0.88 ± 0.02 c
2019	GM	C	W	22.57 ± 0.45 a	10.49 ± 0.20 b	8.63 ± 0.14 b	2.57 ± 0.04 b	3.07 ± 0.06 a	75.51 ± 7.94 a	0.87 ± 0.01 c
2019	GM	T	N	18.73 ± 0.25 c	7.44 ± 0.27 d	5.28 ± 0.12 d	2.37 ± 0.15 b	2.9 ± 0.00 b	26.71 ± 1.89 c	1.12 ± 0.02 a
2019	GM	T	S	20.12 ± 0.26 b	8.24 ± 0.12 c	6.20 ± 0.10 c	2.26 ± 0.09 b	2.97 ± 0.06 b	32.92 ± 0.77 c	1.02 ± 0.01 b
2019	LE	C	NW	15.94 ± 0.14 d	5.96 ± 0.09 c	4.22 ± 0.07 c	1.82 ± 0.03 c	2.87 ± 0.06 c	40.93 ± 1.29 c	1.20 ± 0.01 a
2019	LE	C	SE	16.65 ± 0.30 c	6.24 ± 0.25 c	4.53 ± 0.17 c	1.83 ± 0.10 c	2.90 ± 0.00 c	42.85 ± 1.04 c	1.18 ± 0.02 a
2019	LE	T	NE	24.11 ± 0.51 b	13.33 ± 0.30 b	10.28 ± 0.29 b	4.06 ± 0.20 b	3.17 ± 0.06 b	182.57 ± 19.8 b	0.77 ± 0.01 b
2019	LE	T	SW	28.22 ± 0.25 a	17.33 ± 0.6 a	13.9 ± 0.61 a	5.08 ± 0.07 a	3.27 ± 0.06 a	304.90 ± 33.19 a	0.67 ± 0.01 c
2019	LS	C	NW	19.19 ± 0.04 c	8.03 ± 0.04 c	5.92 ± 0.07 c	2.38 ± 0.01 c	2.93 ± 0.06 ab	63.32 ± 2.25 c	1.00 ± 0.00 b
2019	LS	C	SE	18.76 ± 0.12 c	7.41 ± 0.07 c	5.57 ± 0.06 c	2.19 ± 0.02 c	3.00 ± 0.00 a	56.09 ± 3.99 c	1.06 ± 0.01 a
2019	LS	T	NE	22.06 ± 0.67 b	10.39 ± 0.51 b	8.11 ± 0.44 b	2.69 ± 0.14 b	2.90 ± 0.00 b	113.60 ± 1.72 b	0.86 ± 0.02 c
2019	LS	T	SW	25.26 ± 0.68 a	13.91 ± 0.86 a	11.15 ± 0.79 a	3.35 ± 0.15 a	3.00 ± 0.00 a	171.79 ± 10.11 a	0.75 ± 0.02 d
2020	GM	C	E	17.51 ± 0.08 c	6.98 ± 0.11 a	6.95 ± 0.10 a	0.93 ± 0.07 b	3.07 ± 0.06 b	54.58 ± 2.04 b	1.14 ± 0.01 b
2020	GM	C	W	18.77 ± 0.13 a	7.18 ± 0.26 a	7.09 ± 0.26 a	0.69 ± 0.02 c	3.20 ± 0.00 a	73.13 ± 2.16 a	1.08 ± 0.02 c
2020	GM	T	N	17.79 ± 0.06 c	7.13 ± 0.00 a	6.42 ± 0.05 b	1.42 ± 0.05 a	3.00 ± 0.00 c	38.11 ± 0.75 c	1.17 ± 0.00 a
2020	GM	T	S	18.24 ± 0.37 b	6.88 ± 0.15 a	6.78 ± 0.22 a	0.93 ± 0.02 b	3.10 ± 0.00 b	36.37 ± 2.24 c	1.17 ± 0.02 a
2020	LE	C	NW	14.53 ± 0.13 d	4.65 ± 0.09 d	4.09 ± 0.05 d	0.98 ± 0.05 c	2.90 ± 0.00 b	27.57 ± 1.1 c	1.42 ± 0.01 a
2020	LE	C	SE	15.79 ± 0.27 c	5.57 ± 0.16 c	4.89 ± 0.14 c	1.18 ± 0.05 b	2.90 ± 0.00 b	30.05 ± 1.91 c	1.32 ± 0.02 b
2020	LE	T	NE	17.72 ± 0.11 b	9.61 ± 0.22 b	8.34 ± 0.24 b	2.23 ± 0.03 a	2.97 ± 0.06 a	76.95 ± 2.98 b	1.02 ± 0.01 c
2020	LE	T	SW	19.19 ± 0.44 a	11.15 ± 0.66 a	10.00 ± 0.59 a	2.37 ± 0.14 a	3.00 ± 0.00 a	119.24 ± 6.2 a	0.95 ± 0.02 d
2020	LS	C	NW	15.58 ± 0.21 d	5.21 ± 0.15 d	4.81 ± 0.15 d	0.95 ± 0.03 b	2.90 ± 0.00 b	45.60 ± 3.70 c	1.29 ± 0.02 a
2020	LS	C	SE	16.73 ± 0.34 c	5.98 ± 0.23 c	5.60 ± 0.22 c	1.05 ± 0.05 b	3.00 ± 0.00 a	52.65 ± 2.97 c	1.22 ± 0.02 b
2020	LS	T	NE	18.77 ± 0.24 b	10.82 ± 0.13 b	9.70 ± 0.11 b	2.16 ± 0.03 a	2.87 ± 0.06 b	98.13 ± 6.48 b	0.96 ± 0.01 c
2020	LS	T	SW	20.71 ± 0.43 a	13.05 ± 0.74 a	12.25 ± 0.66 a	2.27 ± 0.12 a	2.90 ± 0.00 b	136.67 ± 15.3 a	0.89 ± 0.02 d

## Supplementary Materials

The following are available online at https://www.mdpi.com/article/10.3390/foods10112682/s1, Table S1: Summarized weather conditions for 2019–2021. Figure S1: Mean air temperature (solid line) and daily rainfall (black bars) during vegetation periods (1 April to 1 October) 2019, 2020 and 2021 in Geisenheim (Rheingau, Germany). Table S2: List of standards used for calibration of the UHPLC and determination of grape skin phenolics. Table S3: Canopy density parameters for experimental sites Geisenheimer Rothenberg (= GM), Lorcher Eisersgrube (= LE) and Lorcher Sesselberg (= LS), separated by treatment (control = C, terraced vineyard = T), exposition and different grapevine growth stages in 2019 and 2020. Table S4: Relative cumulative solar radiation measurements (Whm^−2^) normalized by global radiation data of local weather stations, separated by year for each site, treatment (C = control; T = terraced vineyard), exposition and development stage. Table S5: Amino acid concentration (mean ± standard deviation; mg L^−1^ g^−1^) of Riesling juice obtained from harvest-ripe berries in 2019 and 2020, separated by canopy sides of each treatment. Table S6: Share of single amino acid concentration on total amino acid concentrations (%) of Riesling juice obtained from harvest-ripe berries in 2019 and 2020, separated by canopy sides of each treatment. Table S7: Polyphenolic content (mean ± standard deviation; µg g^−1^ berry fresh weight) of Riesling berry skins obtained from harvest-ripe berries in 2019 and 2020, separated by canopy sides of each treatment.
